# MicroRNAs in Basolateral Amygdala Associated with Stress and Fear Memories Regulate Rapid Eye Movement Sleep in Rats

**DOI:** 10.3390/brainsci11040489

**Published:** 2021-04-12

**Authors:** Nagaraja S. Balakathiresan, Manish Bhomia, Min Zhai, Brook L. W. Sweeten, Laurie L. Wellman, Larry D. Sanford, Barbara Knollmann-Ritschel

**Affiliations:** 1 Department of Pathology, Uniformed Services University of the Health Sciences, Bethesda, MD 20814, USA; manish.bhomia.ctr@usuhs.edu (M.B.); min.zhai.ctr@usuhs.edu (M.Z.); barbara.knollmann-ritschel@usuhs.edu (B.K.-R.); 2 Sleep Research Laboratory, Center for Integrative Neuroscience and Inflammatory Diseases, Department of Pathology and Anatomy, Eastern Virginia Medical School, Norfolk, VA 23507, USA; bwilliams424@gmail.com (B.L.W.S.); WellmaLL@evms.edu (L.L.W.); SanforLD@evms.edu (L.D.S.)

**Keywords:** microRNA, REM sleep, traumatic stress, resilient, vulnerability, fear memory, basolateral amygdala

## Abstract

Stress-related sleep disturbances are distressing clinical symptoms in posttraumatic stress disorder patients. Intensely stressful events and their memories change rapid eye movement (REM) sleep in animal models. REM sleep varies with individual differences of stress resilience or vulnerability. The basolateral amygdala (BLA) is a primary mediator of the effects of stress and fear memories on sleep. However, the molecular mechanisms in BLA regulating the effects of fear conditioning, shock training (ST) and context re-exposure (CTX) on REM sleep are not well known. MicroRNAs (miRNAs) are small, non-coding RNAs and posttranscriptional gene regulators of diverse biological processes. The aim of this study is to investigate ST- and CTX-altered miRNAs in the BLA of resilience and vulnerable animals and on REM sleep regulation. MiRNAs expression profiles in BLA were generated following ST and CTX using the Taqman Low Density rodent microRNA array. The altered BLA miRNAs expression and REM sleep reduction observed in ST and CTX vulnerable animals. AntagomiR-221 microinjection into BLA for one of the upregulated miRNAs, miR-221 in BLA, attenuated the REM sleep reduction. This study suggests that miRNAs in the BLA may play a significant role in mediating the effects of stress and fear memories on REM sleep.

## 1. Introduction

Posttraumatic stress disorder (PTSD) is a neuropsychiatric disorder characterized by re-experiencing a traumatic event, avoidance of traumatic cues, and hyperarousal. It has a lifetime prevalence of 7–8%, and sleep disturbance is one of its most distressing symptoms [1,2]. Stress-related sleep disturbances are highly prevalent in military personnel and veterans, and they can be a significant factor in the diagnosis of PTSD [3]. Studies conducted on US Navy and Marine Corps service members indicated that sleep deprivation was a primary mediating factor in development of PTSD [1]. This finding correlates with various studies that have implicated sleep disturbances, both before and after stressful events, in the development of PTSD and other psychopathology [4,5,6]. Mental health consequences result in a high prevalence of PTSD symptoms associated with poor sleep quality for the public [7,8,9,10]. Disturbances in rapid eye movement (REM) sleep are of particular interest in PTSD. While the precise role of REM sleep in PTSD has not been elucidated, reduced and fragmented REM sleep is associated with both the formation and progression of symptoms in PTSD [11,12,13]. Fear conditioning has a significant impact on sleep. One of the prominent effects of fear on sleep can be reduced REM sleep. The basolateral nucleus of the amygdala (BLA), a region critical for fear memory, appears to be an important site in the brain for mediating the effects of stress on sleep and storing fearful memories and regulating their effects on sleep [14,15,16,17,18,19,20].

In animal models, intense stressful events and memories of those events can be followed by either decreases or increases in sleep, especially REM sleep [19,21,22,23]. REM sleep is implicated in the processing of emotional events, suggesting that these differences may reflect variability in coping associated with individual differences in resilience (Res) and vulnerability (Vul) to stress [19]. Functional studies have demonstrated that the BLA is a significant mediator of the effects of stress and stressful memories on sleep and that it regulates differences in REM sleep in Res and Vul rats [19,20,21,22]. However, BLA’s molecular mechanisms that regulate the effects of fear conditioning (shock training—ST) and fearful memories induced by context re-exposure (CTX) on REM sleep are virtually unknown.

MicroRNAs (miRNAs) are small, 19–28 nucleotides in length, endogenous non-coding RNA molecules. MiRNAs regulate gene expression at post-transcriptional levels, either by translational repression or mRNA degradation by binding to complementary sequences in mRNA [24,25]. MiRNAs are believed to control more than 60% of the protein-coding genes in humans and are involved in diverse biological processes of various diseases and disorders, including neuropsychiatric disorders [24,26,27,28,29,30]. Studies on Gulf war veterans with PTSD indicated that 190 miRNAs were significantly altered in peripheral blood mononuclear cells (PBMC) and that the altered miRNAs had involvement in immune system pathways [31,32]. Other studies on stress, glucocorticoids, and mood stabilizers showed altered levels of miRNAs in patients indicating that they might play potential roles in pathophysiology and therapeutics of mental diseases and disorders [33,34,35]. Li et al. reported that early adolescence acute traumatic stress could cause permanent changes in neural networks in an animal model [36]. The plausibility of using circulating miRNAs as biomarkers of PTSD has been supported by an examination of the expression of serum and amygdala miRNAs in a learned helplessness stress animal model of PTSD [29]. Analysis of sub-acute period post-stress miRNAs in the amygdala showed a global increase in miRNA expression. It revealed a substantial alteration of the post-transcriptional machinery involved in the consolidation and long-term stability of fear memories [37,38]. Therefore, the present study’s primary objective was to build on these findings and determine whether the expression of miRNAs in BLA varied in response to stress and fearful memories and their effects on REM sleep.

We first assessed miRNA expression in BLA following ST in a well-established animal stress model that produces differential REM sleep responses in putative Res and Vul rats [19]. Next, we determined miRNAs altered in fear memory in rats where BLA was inactivated with the GABA_A_ agonist, muscimol (MUS) before ST, the period when fear is learned [22]. We measured the REM sleep and the miRNAs in putative Res and Vul rats’ post-CTX. Finally, to determine whether differentially expressed BLA miRNAs have a role in regulating distinct effects of stress and fear memories on REM sleep, we administered an antagomir to reduce miR-221, one of the upregulated fear associated miRNAs prior to ST. The results showed that pre-ST antagomir-administration into the BLA prevented reductions in REM sleep after ST and CTX and reduced behavioral fear as indicated by freezing.

## 2. Materials and Methods

*Animals*: All animal experiments were conducted at Eastern Virginia Medical School (EVMS) using 90-day-old, male, outbred, Wistar rats purchased from Harlan Laboratories (Frederick, MD). The rats were kept individually in polycarbonate cages and given ad lib access to food and water. The rooms were maintained with 12:12 light–dark and ambient room temperature at 24.5 ± 0.5 °C, as previously reported [19].

*Surgery*: The rats were anesthetized and implanted surgically with electroencephalogram (EEG) and electromyography (EMG) electrodes for recording sleep [19]. Some rats were also implanted with bilateral guide cannulae (26 ga.) with their tips aimed 1.0 mm above BLA (A 2.6, ML ± 4.8, DV 8) and with intraperitoneal temperature recorders (SubCue Dataloggers, Canadian Analytical Technologies, Inc. Calgary, Alberta, Canada) for recording core body temperature and stress-induced hyperthermia (SIH) as previously described [20].

*Training and Procedures*: After surgery, the rats were allowed to recover, and baseline sleep was recorded. Afterward, they were randomly assigned to serve as non-stressed, home cage controls (HC), or they received footshock training (ST; 20 trials, 0.8 mA, 0.5 s, 1 min intervals). Each ST session lasted 30 min. During this procedure, individual rats were placed in shock chambers (Coulbourn Habitest cages equipped with grid floors (Model E10–18RF) housed in Coulbourn Isolation Cubicles (Model H10–23)) and allowed to freely explore for 5 min. Over the next 20 min, they were presented with 20 footshocks (0.8 mA, 0.5 s duration) at 1.0 min intervals. Shock was produced by Coulbourn Precision Regulated Animal Shockers (Model E13–14) and presented via the grid floor of the shock chamber. Five min after the last shock, the rats were returned to their home cage for sleep recording. The shock chamber was thoroughly cleaned with diluted alcohol (70% EtOH) following each session. Each session was videotaped using mini-video cameras (Weldex, WDH-2500BS, 3.6 mm-lens) attached to the center of the ceiling of the shock chamber for subsequent visual scoring of freezing. The ST group was further divided into two subgroups. One subgroup presented with ST alone and based on whether REM sleep was significantly decreased or not decreased compared to baseline. During the first 2 h following ST, the rats were then designated as vulnerable (Vul) or resilient (Res). The rats were sacrificed at 2 h after ST (ST-Vul; *n* = 5; 50% and ST-Res; *n* = 5; 50%).

**Figure 1:**
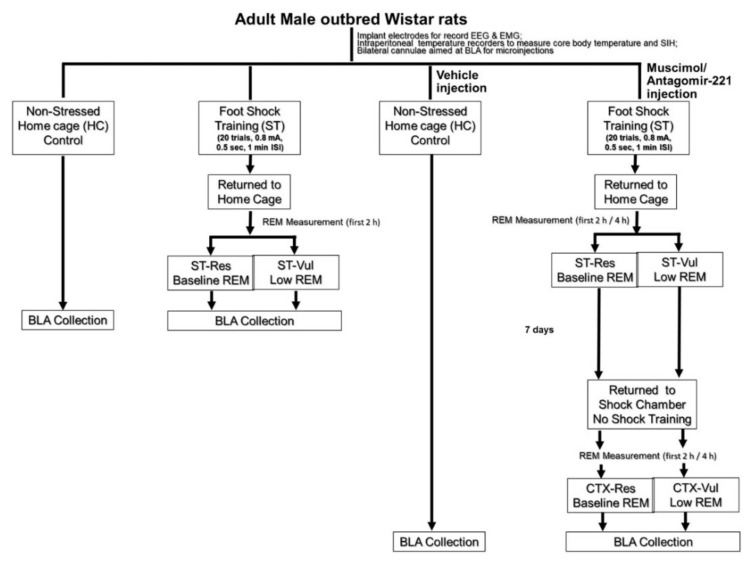
Schematic diagram of the experimental design of the animal model of posttraumatic stress disorder (PTSD) for examining fear-conditioned changes in rapid eye movement (REM) sleep and microRNA expression studies in basolateral amygdala (BLA).

Another subgroup received a 0.5-µL microinjection into BLA with the gamma-aminobutyric acid receptor (GABA_A_) agonist, muscimol (MUS; 1.0 uM; 5-aminomethyl-3-hydroxyisoxazole, Sigma–Aldrich, St. Louis, MO, USA) prepared with RNAse free water, 30 min prior to ST. MUS preparation and the procedure for microinjections were followed, as mentioned earlier [22]. Rats were returned to their home cages after the ST procedure and REM sleep was recorded. Based on the amount of REM sleep compared to baseline during the first 2 h following ST, the rats were then designated as putative Vul or Res. Seven days after ST, rats were placed back into the shock chambers and allowed to explore freely for 30 min where no shock (CTX) was presented. This CTX was used to test the effects of fear memory on REM sleep [19]. The rats were sacrificed at 2 h post-CTX (CTX-Vul; *n* = 5; 56% and CTX-Res; *n* = 4; 44%). HC rats (*n* = 6) were sacrificed at the same circadian time as the ST and CTX rats (Figure 1).

For antagomir studies, Wistar rats (*n* = 28) were separated into control and antagomir (Ant) groups. The Ant group (*n* = 7) received bilateral microinjections into BLA of an antagomir for miR-221 (rno-miR-221-3p; MIMAT0000890). An amount of 5.0 nmol of antagomiR-221 was prepared in 0.5 µL of RNase free water (Veh) as recommended by the supplier (GE Dharmacon). Control animals (*n* = 21) were microinjected with the Veh alone. Sixteen h later, the rats were trained with ST as described above. In preclinical animal models, the pharmacokinetics study LNA-i-miR-221 is detectable up to 7 days after a single injection dose of 25 mg/kg in brain tissues. Further, the in situ hybridization (ISH) analyses in the brain suggest that the 13-mer LNA-i-miR-221 does not cross the brain’s normal blood brain barrier (BBB) [39]. We administered the antagomir-221 directly into BLA 16 h before conditioning should be effective in functional inhibition of miR-221. Based on the amount of REM sleep compared to baseline during the first 4 h following ST, the Veh rats were then designated as Vul (*n* = 11; 52%) and Res (*n* = 10; 48%). Seven days after ST, the rats were returned to shock chamber for 30 min but did not receive a shock (CTX). Sleep was recorded in both Veh (Vul and Res) and Ant treated animals during the first 4 h after CTX and the amount of REM sleep was compared to baseline sleep before ST. Freezing and core body temperature were also examined as described previously [20].

*Sleep recording and measurement*: Sleep recording and sleep data collection were performed as previously described [19]. Briefly, sleep was recorded for each animal by placing on it a rack outfitted for electrophysiological recording and a lightweight and a shielded cable was connected to the miniature plug on the rat’s head. The EEG and EMG signals and were processed by a Grass, Model 12 polygraph equipped with model 12A5 amplifiers and routed to an Analog to Digital (A/D) board (Model USB-2533, Measurement Computing) housed in a personal computer. The signals were digitized at 256 Hz and collected in 10 s epochs using the SleepWave (Biosoft Studio) data collection program. The sleep data analysis was carried out by trained observers visually scoring the computerized EEG and EMG records [19]. Data were collected up to 4 h (for baseline) and for 2 or 4 h after ST and CTX (depending on group) and compared to time-matched baseline recordings. Baseline recordings were obtained prior to ST in animals that were otherwise undisturbed in their home cages.

*Tissue collection*: The BLA was collected 2 h following ST or, in the pre-ST MUS administered group, 2 h following CTX. HC rats were sacrificed at the same circadian time to collect BLA (Figure 1). The collected BLA tissue was immediately frozen at −80 °C for miRNA analysis.

*Total RNA isolation*: Frozen tissue samples were thawed on ice, and the total RNA was isolated using the RNeasy Mini Kit (Qiagen, Valencia, CA, USA), according to the manufacturer’s instructions. The purity of the RNA was assessed by the ratio of absorbance at 260/280 nm using a Nanodrop 2000 spectrophotometer (Thermo Scientific Inc. Pittsburgh, PA). Small RNA quantity and the quality, such as the integrity of the total RNA, was tested by a Bioanalyzer (Agilent Technologies, Inc., Santa Clara, CA, USA).

*Examination of miRNA expressions*: Reverse transcription (RT) was performed with TaqMan microRNA RT Kits (Life Technologies, Carlsbad, CA, USA) using total RNA with Megaplex RT primer pools. The RT product was pre-amplified, and the cycles and conditions were followed according to the manufacturer’s protocol (Life Technologies, Carlsbad, CA, USA). The undiluted pre-amplification products were loaded into each row of the 384-well TaqMan^®^ Low Density rodent MicroRNA Array (TLDA) Rodent Set v3.0 as described earlier [29]. The PCR reaction was carried out at default thermal-cycling conditions in an AB7900HT Fast Real-Time PCR System (Applied Biosystems, Life Technologies, Foster City, CA, USA).

*MiRNA expression analysis*: MiRNA expression profile raw data, i.e., Cycle threshold (Ct) values, were analyzed using StatMiner^®^ (Integromics) software to identify significantly altered miRNAs. For relative quantification of miRNAs between HC and ST or CTX groups, the following steps were performed in the StatMiner software suite. Quality control of biological replicates was carried out for the selection of most stabilized endogenous control. MiRNA expressions with Ct values below 35 cycles were only selected for the detection of expression in all biological replicates of calibrator and target. Statistically significant miRNAs were selected based on *p*-values lower than 0.05.

*Ingenuity pathway analysis*: Targets of altered miRNA were identified using the miRNA target filter application available in the Ingenuity Pathway Analysis (IPA) program (QIAGEN Redwood City, CA, USA). Disease and disorder categories were identified with filtered experimentally predicted target mRNAs with Bio function analysis in IPA. A Custom miRNA–mRNA target network was built with the "My Pathways" application in IPA using ST- or CTX Vul-altered miRNAs and the genes associated with fear memory and sleep disorder in IPA database search results.

*Statistical analysis*: Sleep and temperature data were analyzed with two-way between factor ANOVAs across Group (Vul, Res, and HC) and Time (Baseline and ST). Post hoc comparisons when indicated by a significant ANOVA were conducted with Holm–Sidak tests to maintain *p* < 0.05 across comparisons. For miRNA expression analysis study, we used Benjamini Hochberg False Discovery Rate (FDR) adjustment to create a ΔΔCt value showing any up or downregulation along with unadjusted and adjusted *p* values. Statistically significant miRNAs were selected based on *p* values less than 0.2 for FDR and for the unadjusted *p* value lower than 0.05 [29].

## 3. Results

### 3.1. Selection of Putative Res and Vul Rats for miRNA Profiling Studies

**Figure 2:**
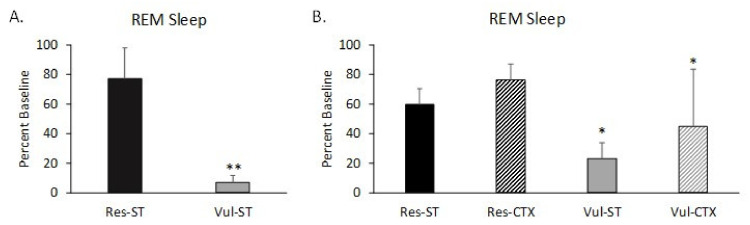
Differential REM sleep responses induced by foot shock stress in outbred Wistar strain rats plotted as percent baseline for the first 2 h after shock training (ST) and context re-exposure (CTX). (**A**) REM in rats sacrificed immediately after ST. Rats vulnerable (Vul) to ST showed significantly reduced REM sleep (Low REM) post ST whereas resilient (Res) rats did not. (**B**) Rats sacrificed after CTX. Res rats did not show significantly reduced REM sleep post-ST or post-CTX whereas Vul rats did. *: *p* < 0.05; **: *p* < 0.01; compared to base (Holm–Sidak test). Data are represented as means ± standard error of the means (SEMs).

The rats were selected based on differences in REM sleep amounts for the first 2 h post ST. The rats which showed significant reduction in REM sleep percentage (low REM). When compared to baseline sleep were selected as putative ST-Vul rats. Putative ST-Res rats showed either no decrease in REM sleep or increase in REM sleep (high REM) compared to baseline sleep (Figure 2A). When ST-Vul rats were re-exposed to the fear context, still they continue to exhibit significant reductions in REM sleep percentage when compared to baseline sleep (CTX-Vul), whereas CTX-Res rats did not show significantly reduced REM sleep percentage (Figure 2B). This classification resulted in the following groupings: ST-Vul (*n* = 5), ST-Res (*n* = 5), CTX-Vul (*n* = 5), CTX-Res (*n* = 4). HC rats (*n* = 6) never received ST or CTX and were used for comparison.

### 3.2. Differential Expression of BLA miRNAs in ST and CTX

We performed real time PCR for a set of 591 rodent miRNAs (mirbase v15) for BLA samples in each stress and control group using the Taqman low-density array (TLDA) platform. For relative quantitation of miRNAs in BLA samples, we used U6 snRNA as endogenous control for the normalization process. For this, we analyzed the expression levels of the endogenous controls such as U6 snRNA, U87, Y1, snoRNA135, snoRNA202, snoRNA429 and negative control ath-miR159, available in the cards analyzed using the genorm algorithm in the StatMiner software to find a stable endogenous control. This analysis showed a high variability of the expression levels of these endogenous controls between the study groups. Among them only U6 snRNA was amplified in all the samples (highlighted in yellow) and showed the lowest Ct variance between control and ST/CTX vulnerable and resilient samples and selected as endogenous control for our normalization studies (Supplementary Table S1). The numbers of the miRNA that passed the detection criteria were similar among the HC and ST or CTX groups. The number of miRNAs expressed ranged from 204 to 258 (Supplementary Figure S1). To determine whether miRNA expressions in BLA were altered after ST or CTX, miRNA expression of the ST or CTX rats was compared to corresponding miRNA expression in HC. The results showed that 12 miRNAs (6 up- and 6 down-regulated) and 3 miRNAs (all downregulated) were significantly altered in Vul and Res, respectively, in fear-conditioned animals following ST (Table 1). In BLA MUS-injected animals, 19 miRNAs (17 up- and 2 downregulated) and 7 miRNAs (2 up- and 5 down-regulated) were significantly altered in the Vul and Res following CTX, respectively (Table 2).

### 3.3. Biological Processes and Network Analysis of Altered miRNAs

**Figure 3:**
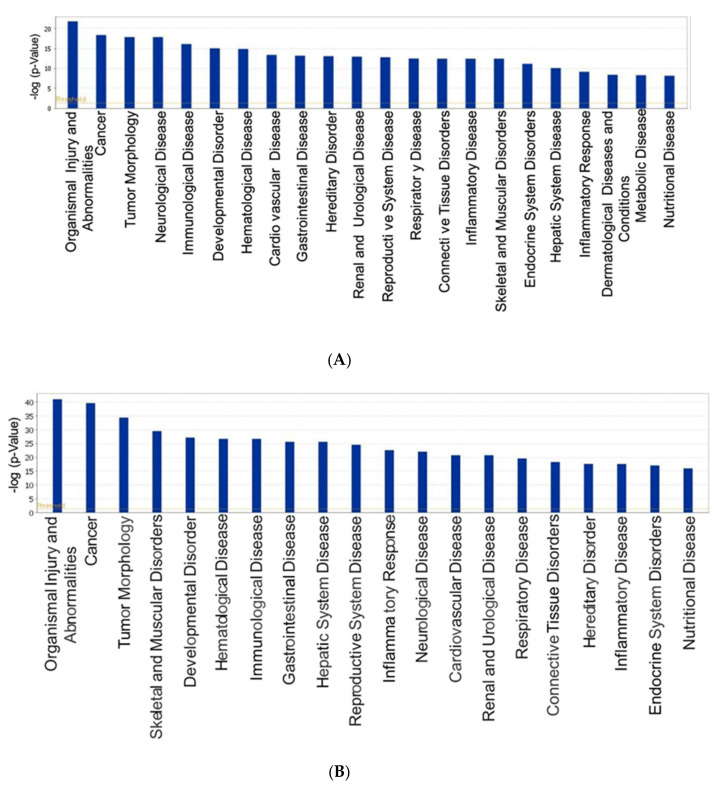

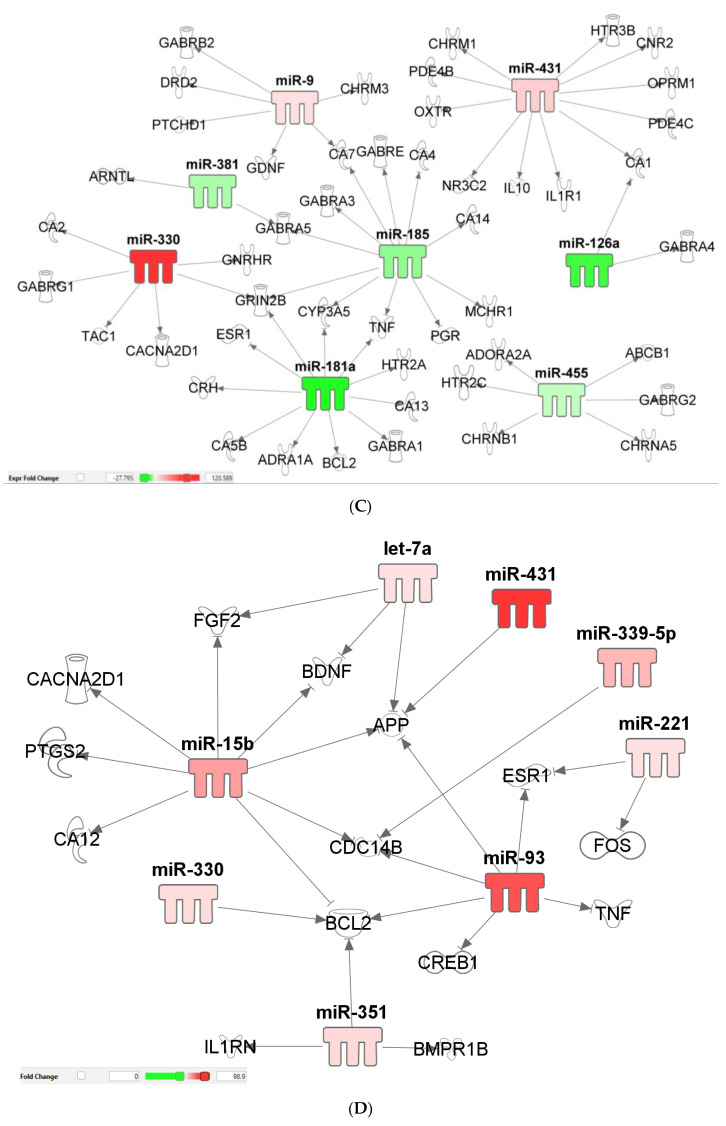
Ingenuity pathway analysis of the rats following ST- and following CTX-altered miRNAs. Diseases and disorder functional analysis of the miRNAs included all the determined experimental targets predicted organismal injury and abnormalities as one of the most significant biological functions in both (**A**) post-ST and (**B**) post CTX. A custom network was built with (**C**) post-ST- and (**D**) post-CTX-altered miRNAs and gene targets commonly implicated in fear and REM sleep disorder showing possible roles of the selected miRNAs in these two activities. The red color indicates the upregulated miRNA expression.

In this study, intense stress produced changes in REM sleep as well as miRNA expression in BLA and was more significant in ST-Vul rats. To identify molecular correlates of altered miRNAs, we used the predicted target genes in IPA to screen biological processes. The miRNA target filter in IPA identified 50 experimentally determined mRNA targets for the ST-Vul-altered miRNAs. Their biological processes predicted organismal injury and abnormalities as the most significant disease and disorder function (Figure 3A). Similarly, after CTX, the miRNA target filter identified 342 experimentally determined targets and their biological processes also indicated organismal injury and abnormalities as the most significant disease and disorder function (Figure 3B). To identify ST altered miRNAs in BLA that are associated with fear and sleep, we constructed networks with mRNA targets related to fear and sleep disturbances in IPA. The networks indicated that miRNAs in Vul rats in both the ST and CTX groups were predicted to mediate both fear and sleep target genes (Figure 3C,D). Network analysis of all upregulated miRNAs in the CTX-Vul group indicated that increased miR-221 act on sleep molecules Fos proto-oncogene, AP-1 transcription factor subunit (Fos) and estrogen receptor 1 (ESR) and may negatively impact REM sleep. Additionally, our studies in a learned helplessness rodent model of PTSD showed increased miR-221 expression and was associated with fear response [29] (Table 2 and Figure 3D). Based on these findings, miR-221 was identified as an appropriate target to improve REM sleep in these animals for the subsequent studies.

### 3.4. Effects of AntagomiR-221 Microinjection on REM Sleep

**Figure 4:**
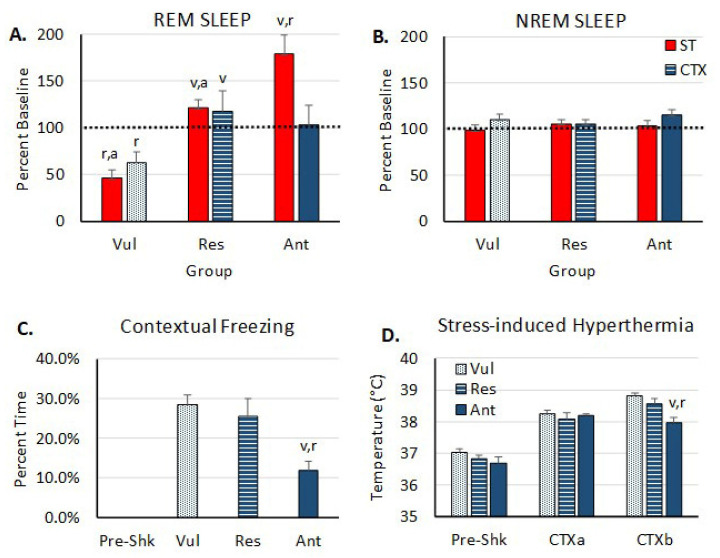
REM (**A**) and NREM (**B**) sleep in the first 4 h after shock training (ST) in vehicle treated vulnerable (Vul, *n* = 11) and resilient (Res, *n* = 10) rats compared to that in rats (*n* = 7) microinjected into BLA with an antagomir for rno-miR-221-3p (Ant). Data are plotted as percentage baseline to adjust for potential cohort differences in baseline REM amounts. Freezing (**C**) and stress-induced hyperthermia (SIH) (**D**) in the fearful context are also shown. Letters (v, r, a) above bars indicate significant differences (*p* < 0.05) to Vul, Res and Ant groups, respectively. Dashed line indicates baseline levels at 100 percent. ST: shock training; CTX: context alone. CTXa and CTXb indicate 15 min intervals for SIH in the 30 min CTX period. Error bars are ± SEM.

Vul rats showed a greater number of altered miRNAs in both the ST and CTX groups. A critical question is whether the differentially expressed miRNAs in BLA play a role in regulating the distinct effects of stress and fear memories on REM sleep in Res and Vul rats. To obtain the answer, we selected miR-221, which has a predicted association to fear (Figure 4D) to investigate further. Compared to baseline levels of REM sleep, rats that received pre-ST antagomir-221 microinjections into BLA exhibited a 78.8 ± 18.9 (mean ± SEM) percent increase in REM sleep in the first 4 h after ST. This increase was significant compared to vehicle treated (Veh) rats that exhibited either the Vul or Res phenotype (Figure 4A). For this study, the Veh rats were designated as Vul and Res based on the amount of REM sleep compared to baseline during the first 4 h following ST. Subsequent exposure of these animals to CTX was followed by a return to baseline levels of REM sleep. We also measured the NREM sleep in these animals; however, it was not altered across group or condition (Figure 4B). Pre-ST antagomir-221 microinjections into BLA reduced freezing compared to control Vul and Res rats during re-exposure to CTX alone (Figure 4C). There was also a reduction in SIH in antagomir-221 treated rats in the second 15 min in CTX (Figure 4D).

## 4. Discussion

Our primary aim of this study was to determine the miRNAs in BLA that regulate the effects of stress on sleep and the formation of memories that can disturb sleep in the future. For this, we first performed a study to identify miRNA expression in the BLA associated with the effects of stress or fear conditioning on REM sleep. For our study, we used a well-established animal model of fear conditioning that produces different REM sleep responses in putative Res and Vul rats to identify miRNAs that regulate differential sleep responses to stress and fear memory [19]. As shown in Figure 2A,B, Res animals showed normal or increased REM sleep after stress and Vul animals show decreased REM sleep. MiRNA expression in Res and Vul rats also showed directionally different alterations in response to ST with a greater number of differentially expressed miRNAs being found in rats showing marked reductions in post-ST REM sleep. The substantial differences in miRNA data between Res and Vul rats complement previous findings that individual, outbred Wistar strain rats can show marked differences in post-stress sleep even though they experienced an identical stressor and show similar indices of fear memory (i.e., behavioral freezing) and the peripheral stress response (i.e., SIH) [19]. They also support prior findings that the BLA plays a major role in regulating stress-induced alterations in REM sleep.

The BLA is an essential region for forming fear memory and mediating fear behavior and it has a critical role in determining the effects of fear memories on sleep [19]. Previously, we found that inactivating the BLA with MUS before ST blocked effects of footshock stress effects and fear memory on REM sleep [22]. In this study we sought to use MUS to assess the effects of blocking BLA on fear memory-induced alterations in miRNAs. However, we saw no effects of blocking BLA on fear memory-induced alterations in this study. This could have resulted from the fact that the original study did not address potential individual differences between Res and Vul rats [22]. It could also result from the fact that we only evaluated 2 h or post-CTX REM in determining differences in Res and Vul rats; we have typically examined 4 h of sleep recording, and the first 2 h is more variable. Even with these limitations, we found differential miRNAs expression in BLA of CTX-Res and CTX-Vul rats that showed increased/normal amounts REM sleep and reduced REM sleep, respectively. CTX-Vul animals showed reduced REM sleep as well as a greater number of differentially expressed miRNAs compared to CTX-Res rats. These findings demonstrate the need to look at additional time periods for both REM and miRNA expression changes in the future.

We observed a significant number of miRNAs differentially expressed after ST compared to CTX. These results are consistent with prior studies that have focused primarily on the number of amygdala miRNAs altered when mice subjected to physical or psychological stress and associations were made between altered miRNAs, fear memory, depression, and resilience [40,41]. To understand the molecular correlates of the altered miRNAs, we performed biological processing and network analysis in IPA. Altered miRNAs in ST-Vul and CTX-Vul rats and their experimentally predicted target biological processes in IPA showed organismal injury and abnormalities as the topmost disease and disorder functional category. Further annotation indicated that organismal injury and abnormalities may involve cell death and apoptosis of brain cells. Our data also correlate with studies demonstrating that stress triggers molecular changes in sleep-related brain regions and is associated with genes involved in cell death and survival. For example, REM sleep disturbances are associated with an increase in cell death and apoptosis in rat brain [24,42]. As shown in Figure 3C,D, the analysis of networks indicates that miRNAs in Vul rats after ST and CTX are predicted to mediate fear and sleep target genes.

In addition to assessing the relationship of miRNA expression in BLA to REM sleep, we also examined the possibility of whether differentially expressed miRNAs in BLA play a role in regulating the distinct effects of stress and fear memories on the sleep in Res and Vul rats. We selected miR-221 because it showed increased expression only in Vul individuals that showed reductions in REM after CTX and because it was upregulated in the sub-acute phase of a learned helplessness rodent model of PTSD [29]. Additionally, evidence from molecular functional network analyses suggests that miR-221 may directly affect stathmin1 (STMN1) regulation, a vital molecule in the amygdala involved in fear conditioning [29,43]. Our molecular functional network construction using CTX miRNAs with fear and sleep disturbance related genes suggests that miR-221 may have a direct role in Fos gene regulation (Figure 3D) which is an increase in the BLA of mice trained with inescapable foot shock [22,44]. Lastly, a recent pilot study in Dutch military members identified circulating serum miR-221 as a potential diagnostic biomarker of PTSD because it correctly distinguished PTSD subjects from controls [45]. These data made miR-221 an ideal candidate to assess local knockdown effects in BLA on stress-related fear memories and sleep. Our data suggest that an antagomir for miR-221 can attenuate the elevated expression of miR-221 and subsequently block reductions of REM sleep after ST, and thus demonstrate that miR-221 is a likely mediator of the differences REM exhibited by Res and Vul rats. In future studies, we plan to thoroughly assess the role of miR-221 and other highly expressed miRNAs in regulating stress-induced changes in REM sleep.

## 5. Conclusions

The data demonstrate that directionally different alterations in REM following stress/context re-exposure are associated with differential miRNA expression in BLA, a region critical for forming fear memory and mediating its effect on sleep. The data also show that it will be valuable to incorporate evaluations of resilience and vulnerability to stress/context re-exposure into studies of miRNAs in animal models of PTSD. Future studies on the role of differentially expressed miRNA(s), their gene targets, and proteins may ultimately lead to a novel therapeutic approach to reverse the harmful effects of traumatic stress on sleep.

Limitations: our study provides novel insights into the role of miRNAs in REM sleep regulation, however there are few limitations of this study. When we conducted this study, we had a limitation of selecting the animals prior to administering the antagomir. We now know that the rats differentially respond to open field stress in much the same way they do to shock training and that baseline brain-derived neurotrophic factor (BDNF) levels may predict sleep responses [46]. We could use these measures for predicting Res and Vul responses in future studies. We could also block miRNA 221 after selection but prior to re-exposure to the fearful context to assess its role in regulating fear-conditioned REM responses in Res and Vul rats. However, the current study is limited to conclusions that can be drawn from the increased REM aftershock training and the baseline levels of REM after the fearful context. The effect of miRNAs on REM sleep levels without prior inactivation with muscimol is not studied in this manuscript. These limitations will be addressed in our future investigations.

## Figures and Tables

**Table 1:** Traumatic stress-altered miRNAs of post ST-Vul and ST Res compared to home cage control (HC) rats. MIMAT-miRBase mature sequence accession number.

S#	Detector	miRBase Accession	Fold Change	*p* Value
**Stress Vulnerable (ST-Vul)**				
1	miR-503	MIMAT0004790	160.43	0.003
2	miR-330	MIMAT0000568	120.59	0.0005
3	miR-331	MIMAT0004643	43.96	0.02
4	miR-431	MIMAT0001626	28.87	0.04
5	miR-136	MIMAT0004532	9.93	0.02
6	miR-9	MIMAT0000781	9.69	0.01
7	miR-455	MIMAT0003742	−7.54	0.03
8	miR-203	MIMAT0000876	−8.24	0.05
9	miR-381	MIMAT0003199	−10.36	0.03
10	miR-185	MIMAT0000862	−13.72	0.05
11	miR-126	MIMAT0000831	−23.55	0.02
12	miR-181a	MIMAT0000858	−27.80	0.04
**Stress Resilient (ST-Res)**				
1	miR-126	MIMAT0000831	−18.49	0.03
2	miR-185	MIMAT0000862	−23.94	0.01
3	miR-344	MIMAT0000592	−119.02	0.008

**Table 2:** Traumatic stress-altered miRNAs of post CTX-Vul and CTX-Res compared to HC rats. MIMAT-miRbase mature sequence accession number.

S#	Detector	miRBase Accession	Fold Change	*p* Value
**Context Re-exposure Vulnerable (CTX-Vul)**				
1	miR-345	MIMAT0000595	105.86	0.003
2	miR-24	MIMAT0005441	103.29	0.0004
3	miR-431	MIMAT0001626	98.90	0.01
4	miR-93	MIMAT0000817	83.27	0.004
5	miR-490	MIMAT0012823	55.29	0.01
6	miR-382	MIMAT0003201	49.48	0.01
7	miR-15b	MIMAT0000784	46.86	0.003
8	miR-17	MIMAT0000786	44.21	0.02
9	miR-339	MIMAT0004648	43.93	0.02
10	miR-187	MIMAT0000864	35.86	0.02
11	miR-339	MIMAT0000583	34.64	0.01
12	miR-20b	MIMAT0003211	26.60	0.03
13	miR-331	MIMAT0004643	22.05	0.03
14	miR-351	MIMAT0000609	17.87	0.04
15	miR-330	MIMAT0004641	16.18	0.05
16	let-7a	MIMAT0005439	14.00	0.03
17	miR-221	MIMAT0000890	10.11	0.03
18	miR-24	MIMAT0000218	−12.57	0.05
19	miR-28	MIMAT0004661	−193.43	0.002
**Context Re-exposure Resilient (CTX-Res)**				
1	miR-345	MIMAT0000595	36.07	0.02
2	miR-431	MIMAT0001626	23.70	0.05
3	miR-296	MIMAT0000898	−14.47	0.02
4	miR-761	MIMAT0012853	−19.23	0.04
5	miR-24	MIMAT0000218	−40.31	0.01
6	miR-181a	MIMAT0000858	−45.72	0.02
7	miR-28	MIMAT0004661	−15351.58	0.0001

## Supplementary Materials

The following are available online at https://www.mdpi.com/article/10.3390/brainsci11040489/s1, Figure S1: MiRNAs that show Ct < 35 were considered as expressed. The number of miRNAs ranged from 204 to 258 with minimum and maximum numbers detected in ST-Res and CTX-Vul. The SD ranged from 18 to 73, the maximum being in ST-Res and the minimum in HC. The values are presented as Mean + SD of the number of miRNAs detected in each group. Table S1: Ct values for the candidate endogenous controls (U6, snoRNA135, snoRNA202, snoRNA429, U87, Y1) and negative control (ath-miR). StatMiner^®^ Software analysis showed U6 only amplified in all the samples (highlighted in yellow) and the lowest Ct variance between control and ST/CTX Vulnerable and Resilient samples and were chosen as reference genes for data normalization.

## References

[1] Gilbert K.S., Kark S.M., Gehrman P., Bogdanova Y. (2015). Sleep disturbances, TBI and PTSD: Implications for treatment and recovery. Clin. Psychol. Rev..

[2] Maher M.J., Rego S.A., Asnis G.M. (2006). Sleep disturbances in patients with post-traumatic stress disorder: Epidemiology, impact and approaches to management. CNS Drugs.

[3] Moore B.A., Brock M.S., Brager A., Collen J., LoPresti M., Mysliwiec V. (2020). Posttraumatic Stress Disorder, Traumatic Brain Injury, Sleep, and Performance in Military Personnel. Sleep Med. Clin..

[4] Koren D., Arnon I., Lavie P., Klein E. (2002). Sleep Complaints as Early Predictors of Posttraumatic Stress Disorder: A 1-Year Prospective Study of Injured Survivors of Motor Vehicle Accidents. Am. J. Psychiatry.

[5] Lavie P. (2001). Sleep Disturbances in the Wake of Traumatic Events. N. Engl. J. Med..

[6] Bryant R.A., Creamer M., O’Donnell M., Silove D., McFarlane A.C. (2010). Sleep Disturbance Immediately Prior to Trauma Predicts Subsequent Psychiatric Disorder. Sleep.

[7] Huang Y., Zhao N. (2020). Generalized anxiety disorder, depressive symptoms and sleep quality during COVID-19 outbreak in China: A web-based cross-sectional survey. Psychiatry Res..

[8] Liu N., Zhang F., Wei C., Jia Y., Shang Z., Sun L., Wu L., Sun Z., Zhou Y., Wang Y. (2020). Prevalence and predictors of PTSS during COVID-19 outbreak in China hardest-hit areas: Gender differences matter. Psychiatry Res..

[9] Yin Q., Sun Z., Liu T., Ni X., Deng X., Jia Y., Shang Z., Zhou Y., Liu W. (2020). Posttraumatic stress symptoms of health care workers during the corona virus disease 2019. Clin. Psychol. Psychother..

[10] Rajkumar R.P. (2020). COVID-19 and mental health: A review of the existing literature. Asian J. Psychiatry.

[11] Mellman T.A., Kobayashi I., LaVela J., Wilson B., Brown T.S.H. (2014). A Relationship between REM Sleep Measures and the Duration of Posttraumatic Stress Disorder in a Young Adult Urban Minority Population. Sleep.

[12] Ross R.J. (2014). The Changing REM Sleep Signature of Posttraumatic Stress Disorder. Sleep.

[13] Ross R.J., Ball W.A., Dinges D.F., Kribbs N.B., Morrison A.R., Silver S.M., Mulvaney F.D. (1994). Rapid eye movement sleep disturbance in posttraumatic stress disorder. Biol. Psychiatry.

[14] Cousens G., Otto T. (1998). Both pre- and posttraining excitotoxic lesions of the basolateral amygdala abolish the expression of olfactory and contextual fear conditioning. Behav. Neurosci..

[15] Koo J.W., Han J.-S., Kim J.J. (2004). Selective Neurotoxic Lesions of Basolateral and Central Nuclei of the Amygdala Produce Differential Effects on Fear Conditioning. J. Neurosci..

[16] Maren S., Aharonov G., Fanselow M.S. (1996). Retrograde Abolition of Conditional Fear after Excitotoxic Lesions in the Ba-solateral Amygdala of Rats: Absence of a Temporal Gradient. Behav. Neurosci..

[17] Muller J., Corodimas K.P., Fridel Z., LeDoux J.E. (1997). Functional Inactivation of the Lateral and Basal Nuclei of the Amygdala by Muscimol Infusion Prevents Fear Conditioning to an Explicit Conditioned Stimulus and to Contextual Stimuli. Behav. Neurosci..

[18] Sacchetti B., Lorenzini C.A., Baldi E., Tassoni G., Bucherelli C. (1999). Auditory Thalamus, Dorsal Hippocampus, Basolateral Amygdala, and Perirhinal Cortex Role in the Consolidation of Conditioned Freezing to Context and to Acoustic Conditioned Stimulus in the Rat. J. Neurosci..

[19] Wellman L.L., Fitzpatrick M.E., Hallum O.Y., Sutton A.M., Williams B.L., Sanford L.D. (2016). Individual Differences in Animal Stress Models: Considering Resilience, Vulnerability, and the Amygdala in Mediating the Effects of Stress and Conditioned Fear on Sleep. Sleep.

[20] Wellman L.L., Fitzpatrick M.E., Hallum O.Y., Sutton A.M., Williams B.L., Sanford L.D. (2017). The basolateral amygdala can mediate the effects of fear memory on sleep independently of fear behavior and the peripheral stress response. Neurobiol. Learn. Mem..

[21] Wellman L.L., Fitzpatrick M.E., Sutton A.M., Williams B.L., Machida M., Sanford L.D. (2018). Antagonism of corticotropin releasing factor in the basolateral amygdala of resilient and vulnerable rats: Effects on fear-conditioned sleep, temperature and freezing. Horm. Behav..

[22] Wellman L.L., Fitzpatrick M.E., Machida M., Sanford L.D. (2014). The basolateral amygdala determines the effects of fear memory on sleep in an animal model of PTSD. Exp. Brain Res..

[23] Murkar A.L., De Koninck J. (2018). Consolidative mechanisms of emotional processing in REM sleep and PTSD. Sleep Med. Rev..

[24] Balakathiresan N.S., Sharma A., Chandran R., Bhomia M., Zhang Z., Wang K.K.W., Maheshwari R.K., Wang K.K.W., Zhang Z., Kobeissy F.H. (2014). Molecular Mech-anisms and Biomarker Perspective of Micrornas in Traumatic Brain Injury. Biomarkers of Brain Injury and Neurological Disorders.

[25] Brown B.D., Naldini L. (2009). Exploiting and antagonizing microRNA regulation for therapeutic and experimental applications. Nat. Rev. Genet..

[26] Sayed D., Abdellatif M. (2011). MicroRNAs in Development and Disease. Physiol. Rev..

[27] Hrdlickova B., de Almeida R.C., Borek Z., Withoff S. (2014). Genetic variation in the non-coding genome: Involvement of micro-RNAs and long non-coding RNAs in disease. Biochim. Biophys. Acta (BBA) Mol. Basis Dis..

[28] Balakathiresan N.S., Bhomia M., Chandran R., Chavko M., McCarron R.M., Maheshwari R.K. (2012). MicroRNA Let-7i Is a Promising Serum Biomarker for Blast-Induced Traumatic Brain Injury. J. Neurotrauma.

[29] Balakathiresan N.S., Chandran R., Bhomia M., Jia M., Li H., Maheshwari R.K. (2014). Serum and amygdala microRNA signatures of posttraumatic stress: Fear correlation and biomarker potential. J. Psychiatr. Res..

[30] Bhomia M., Balakathiresan N.S., Wang K.K., Papa L., Maheshwari R.K. (2016). A Panel of Serum MiRNA Biomarkers for the Diagnosis of Severe to Mild Traumatic Brain Injury in Humans. Sci. Rep..

[31] Bam M., Yang X., Zumbrun E.E., Zhong Y., Zhou J., Ginsberg J.P., Leyden Q., Zhang J., Nagarkatti P.S., Nagarkatti M. (2016). Dysregulated immune system networks in war veterans with PTSD is an outcome of altered miRNA expression and DNA methylation. Sci. Rep..

[32] Bam M., Yang X., Zumbrun E.E., Ginsberg J.P., Leyden Q., Zhang J., Nagarkatti P.S., Nagarkatti M. (2017). Decreased AGO2 and DCR1 in PBMCs from War Veterans with PTSD leads to diminished miRNA resulting in elevated inflammation. Transl. Psychiatry.

[33] Hunsberger J.G., Fessler E.B., Chibane F.L., Leng Y., Maric A., Elkahloun A.G., Chuang D.-M. (2013). Mood stabilizer-regulated miRNAs in neuropsychiatric and neurodegenerative diseases: Identifying associations and functions. Am. J. Transl. Res..

[34] Jung S.H., Wang Y., Kim T., Tarr A., Reader B., Powell N., Sheridan J.F. (2015). Molecular mechanisms of repeated social defeat-induced glucocorticoid resistance: Role of microRNA. Brain, Behav. Immun..

[35] Ma K., Xu A., Cui S., Sun M.-R., Xue Y.-C., Wang J.-H. (2016). Impaired GABA synthesis, uptake and release are associated with depression-like behaviors induced by chronic mild stress. Transl. Psychiatry.

[36] Li C., Liu Y., Liu D., Jiang H., Pan F. (2016). Dynamic Alterations of Mir-34c Expression in the Hypo-thalamus of Male Rats after Early Adolescent Traumatic Stress. Neural Plast..

[37] Fanselow M.S., LeDoux J.E. (1999). Why We Think Plasticity Underlying Pavlovian Fear Conditioning Occurs in the Basolateral Amygdala. Neuron.

[38] Nader K., Schafe G.E., Le Doux J.E. (2000). Fear Memories Require Protein Synthesis in the Amygdala for Re-consolidation after Retrieval. Nature.

[39] Gallo Cantafio M.E., Nielsen B.S., Mignogna C., Arbitrio M., Botta C., Frandsen N.M., Rolfo C., Tagliaferri P., Tassone P., di Martino M.T. (2016). Pharmacokinetics and Pharmacodynamics of a 13-Mer Lna-Inhibitor-Mir-221 in Mice and Non-Human Primates. Mol. Ther. Nucleic Acids.

[40] Shen M., Song Z., Wang J.H. (2019). Microrna and Mrna Profiles in the Amygdala Are Associated with Stress-Induced De-pression and Resilience in Juvenile Mice. Psychopharmacology.

[41] Sun Y., Lu W., Du K., Wang J.-H. (2019). microRNA and mRNA profiles in the amygdala are relevant to fear memory induced by physical or psychological stress. J. Neurophysiol..

[42] Felmingham K.L., Dobson-Stone C., Schofield P.R., Quirk G.J., Bryant R.A. (2013). The Brain-Derived Neurotrophic Factor Val66Met Polymorphism Predicts Response to Exposure Therapy in Posttraumatic Stress Disorder. Biol. Psychiatry.

[43] Shumyatsky G.P., Malleret G., Shin R.-M., Takizawa S., Tully K., Tsvetkov E., Zakharenko S.S., Joseph J., Vronskaya S., Yin D. (2005). stathmin, a Gene Enriched in the Amygdala, Controls Both Learned and Innate Fear. Cell.

[44] Liu X., Tang X., Sanford L.D. (2003). Fear-conditioned suppression of REM sleep: Relationship to Fos expression patterns in limbic and brainstem regions in BALB/cJ mice. Brain Res..

[45] Snijders C., Krauskopf J., Pishva E., Eijssen L., Machiels B., Kleinjans J., Kenis G., Hove D.V.D., Kim M.O., Boks M.P.M. (2019). Circulating Serum MicroRNAs as Potential Diagnostic Biomarkers of Posttraumatic Stress Disorder: A Pilot Study. Front. Genet..

[46] Sweeten B.L.W., Sutton A.M., Wellman L.L., Sanford L.D. (2019). Predicting Stress Resilience and Vulnerability: Brain-Derived Neurotrophic Factor and Rapid Eye Movement Sleep as Potential Biomarkers of Individual Stress Responses. Sleep.

