# Supplementary material for: MicroRNAs in Basolateral Amygdala Associated with Stress and Fear Memories Regulate Rapid Eye Movement Sleep in Rats
Source: Brain Sci. 2021 Apr 12;11(4):489. doi: 10.3390/brainsci11040489 (PMC8069888; doi:10.3390/brainsci11040489)
Supplement: Supplementary file 1 [file brainsci-11-00489-s001.pdf]

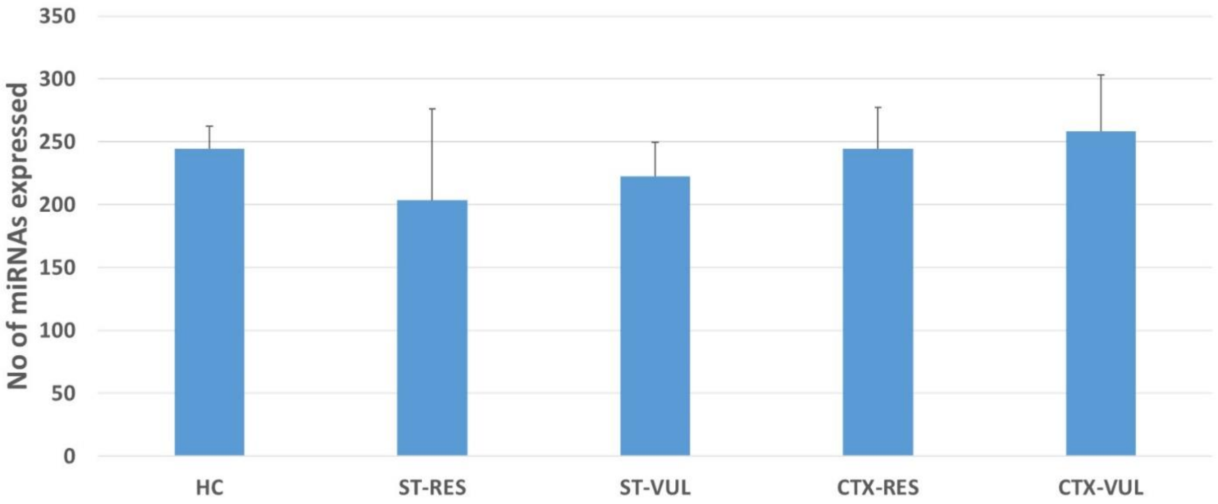

Figure S1. MiRNAs that show Ct ≤35 were considered as expressed. The number of miRNAs ranged from 204 to 258 with minimum and maximum numbers detected in ST-Res and CTX-Vul. The SD ranged from 18 to 73, the maximum being in ST-Res and the minimum in HC. The values are presented as Mean ± SD of the number of miRNAs detected in each group.

Table S1. Ct Values for the candidate endogenous controls (U6, snoRNA135, snoRNA202, snoRNA429, U87, Y1) and negative control (ath-miR). StatMiner® Software analysis showed U6 only amplified in all the samples (highlighted in yellow) and the lowest Ct variance between control and ST/CTX Vulnerable and Resilient samples and was chosen as reference genes for data normalization.

Detector		ath-miR159a-000338	snoRNA135-001230	snoRNA202-001232	snoRNA429-001240	U6 snRNA-001973	U87-001712	Y1-001727
Control	Ct.PSA.WJ40	40	40	40	40	30	31	40
	Ct.PSA.WJ41	40	40	40	40	29	32	40
	Ct.PSA.WJ42	40	33	40	40	27	32	40
	Ct.PSA.WJ43	40	33	40	40	28	33	35
	Ct.PSA.WJ44	40	40	40	40	29	40	40
	Ct.PSA.WJ45	40	40	40	40	24	30	33
ST-Res	Ct.PSB.WJ30	40	40	40	40	30	40	40
	Ct.PSB.WJ33	40	40	40	40	29	40	40
	Ct.PSB.WJ35	40	40	40	40	32	40	40
	Ct.PSB.WJ37	40	40	40	40	26	33	31
	Ct.PSB.WJ38	40	40	40	40	28	33	40
ST-Vul	Ct.PSC.WJ31	40	40	40	40	28	33	31
	Ct.PSC.WJ32	40	40	40	40	34	40	40
	Ct.PSC.WJ34	40	40	40	40	34	40	40
	Ct.PSC.WJ36	40	40	40	40	26	31	40
	Ct.PSC.WJ39	40	40	40	40	26	31	40
CTX-Vul	Ct.PSD.WJ20	40	40	40	40	23	30	31
	Ct.PSD.WJ21	40	40	40	40	33	40	40
	Ct.PSD.WJ24	40	33	40	40	31	40	32
	Ct.PSD.WJ25	40	40	40	40	27	31	40
	Ct.PSD.WJ26	40	40	40	40	30	35	32
CTX-Res	Ct.PSE.WJ22	40	40	40	40	24	31	32
	Ct.PSE.WJ27	40	40	40	40	30	40	38
	Ct.PSE.WJ28	40	40	40	40	31	40	40
	Ct.PSE.WJ29	40	40	40	40	27	35	40
